# Before It Gets Started: Regulating Translation at the 5′ UTR

**DOI:** 10.1155/2012/475731

**Published:** 2012-05-28

**Authors:** Patricia R. Araujo, Kihoon Yoon, Daijin Ko, Andrew D. Smith, Mei Qiao, Uthra Suresh, Suzanne C. Burns, Luiz O. F. Penalva

**Affiliations:** ^1^Greehey Children's Cancer Research Institute, UTHSCSA, San Antonio, TX 78229-3900, USA; ^2^Department of Epidemiology and Biostatistics, UTHSCSA, San Antonio, TX 78229-3900, USA; ^3^Department of Management Science and Statistics, UTSA, San Antonio, TX 78249-0631, USA; ^4^Molecular and Computational Biology, Department of Biological Sciences, USC, Los Angeles, CA 90089-2910, USA; ^5^Department of Cellular and Structural Biology, UTHSCSA, San Antonio, TX 78229-3900, USA

## Abstract

Translation regulation plays important roles in both normal physiological conditions and diseases states. This regulation requires cis-regulatory elements located mostly in 5′ and 3′ UTRs and trans-regulatory factors (e.g., RNA binding proteins (RBPs)) which recognize specific RNA features and interact with the translation machinery to modulate its activity. In this paper, we discuss important aspects of 5′ UTR-mediated regulation by providing an overview of the characteristics and the function of the main elements present in this region, like uORF (upstream open reading frame), secondary structures, and RBPs binding motifs and different mechanisms of translation regulation and the impact they have on gene expression and human health when deregulated.

## 1. Translation Regulation

Gene expression can be modulated at multiple levels from chromatin modification to mRNA translation. Despite the importance of transcriptional regulation, it is clear at this point that mRNA levels cannot be used as a sole parameter to justify the protein content of a cell. In fact, in a recent study from our lab, we determined that a direct correlation between mRNA and protein exists for less than a third of analyzed genes in a human cell line. Moreover, our analysis suggested that translation regulation contributes considerably to the protein variation as several parameters related to translation like 5′ UTR, 3′ UTR, coding sequence length, presence of uORFs and amino acid composition, and so forth showed good correlations with the obtained mRNA/protein ratios [1]. Translation regulation functions as an important switch when rapid changes in gene expression are required in reponse to internal and external stimuli (*PDGF2, VEGF, TGF*β** are examples of genes controlled in such way). Translation regulation also plays a significant role during development and cell differentiation by altering the levels of expression of specific mRNA subsets during a particular time window while the majority of transcripts remain unchanged (reviewed in [2–4]).

**Figure 1:**
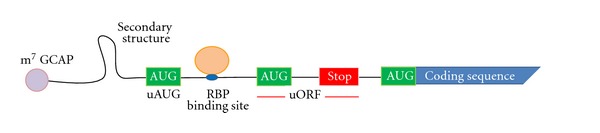
Regulatory elements present in 5′ UTR.

 In this paper, we will focus on the importance of 5′ UTR mediated regulation and the different functional elements present in this region with the exception of IRES which is discussed in a different article of this issue. The main regulatory elements in 5′ UTR are secondary structures (including IRES), binding sites for RNA binding proteins, uAUGs and uORFs (Figure 1).

## 2. 5′ UTR

The average length of 5′ UTRs is ~100 to ~220 nucleotides across species [5]. In vertebrates, 5′ UTRs tend to be longer in transcripts encoding transcription factors, protooncogenes, growth factors and their receptors, and proteins that are poorly translated under normal conditions [6]. High GC content is also a conserved feature, with values surpassing 60% in the case of warm-blooded vertebrates. In the context of hairpin structures, GC content can affect protein translation efficiency independent of hairpin thermal stability and hairpin position [7]. UTRs of eukaryotic mRNAs also display a variety of repeats that include short and long interspersed elements (SINEs and LINEs, resp.), simple sequence repeats (SSRs), minisatellites, and macrosatellites [5].

Translation initiation in eukaryotes requires the recruitment of ribosomal subunits at either the 5′ m7G cap structure. The initiation codon is generally located far downstream, requiring ribosomal movement to this site. This movement appears to be nonlinear for some mRNAs (i.e., ribosomal subunits appear to bypass (shunt) segments of the 5′ UTR as they move in the direction of the AUG). Shunting could allow mRNAs containing uAUGs or hairpin structures to be translated efficiently. Important examples are provided by the cauliflower mosaic virus [8] and adenovirus [9] mRNAs. The mechanism of ribosomal shunting is rather complex requiring mRNA-rRNA base pairing [10].

Genes presenting differences in the 5′ UTR of their transcripts are relatively common. 10–18% of genes express alternative 5′ UTR by using multiple promoters [11, 12] while alternative splicing within UTRs is estimated to affect 13% of genes in the mammalian transcriptome [13]. These variations in 5′ UTR can function as important switches to regulate gene expression. Two important examples are provided by the cancer-related genes *BRCA1* (breast cancer 1) and *TGF-*β** (transforming growth factor *β*). BRCA1 is a tumor suppressor, frequently mutated in breast cancer with functions in cell cycle, apoptosis, and DNA damage repair. BRAC1 produces two different transcripts that derive from two different promoters and therefore display differences in their 5′ UTR. A shorter transcript is expressed in cancerous as well as noncancerous breast tissue and efficiently translated, while a longer transcript is predominantly expressed in breast cancers. The presence of several uAUGs and a more complex structure dramatically affect the translation of this longer transcript. This causes an overall decrease in BRAC1 levels in tumor cells, leading to a relief in growth inhibition [14]. *TGF-*β** is implicated in a large number of processes that include cell proliferation, migration, wound repair, development, tumorigenesis and immunosuppression. There are three known isoforms: *β*1, *β*2, and *β*3. *TGF-*β*3 *produces two alternative transcripts: a 3.5 kb transcript with a very long 5′ UTR (1.1 kb) and a 2.6 kb transcript with a shorter 5′ UTR (0.23 kb). The presence of 11 uORFs in the longer transcript dramatically inhibits its translation while the shorter transcript is efficiently translated [15, 16].

## 3. Regulation by Secondary Structure

Secondary structures can function as major regulatory tools in 5′ UTRs. A correlation with gene function has been suggested; secondary structures have been determined to be particularly prevalent among mRNAs encoding transcription factors, protooncogenes, growth factors, and their receptors and proteins poorly translated under normal conditions. >90% of transcripts in these classes have 5′ UTRs containing stable secondary structures with average free energies less than −50 kcal/mol. 60% of these stable secondary structures are positioned very close to the cap structure [6]. These structures are very effective in inhibiting translation. In fact, a hairpin situated close to the cap with a free energy of −30 kcal/mol would be sufficient to block the access of the preinitiation complex to the mRNA. When located further away in the 5′ UTR, hairpins require a free energy stronger than −50 kcal/mol to be able to block translation [17, 18]. Stable secondary structure can resist the unwinding activity of the helicase elF4A. This effect can be overcome partially by the overexpression of elF4A in partnership with elF4B [19]. mRNAs with a highly structured 5′ UTR like proto-oncogenes and other growth factors use cap-dependent translation initiation. Not surprisingly, the overexpression of components of the translation initiation machinery including elf4E has been linked to tumorigenesis (reviewed in [18, 20]).

 The gene *TGF-*β*1* provides a good example of translation inhibition mediated by secondary structure [21, 22]. An evolutionary conserved motif in the 5′ UTR forms a stable stem loop. However, this structure by itself is not sufficient to block translation. Translation repression of *TGF-*β*1* depends on increased binding of the RNA binding protein YB-1 to the *TGF-*β*1* transcript [23]. It was then proposed that YB-1 binds the 5′ UTR of *TGF-*β*1* with high affinity thanks to its GC content and cooperates with the stem loop to inhibit *TGF-*β*1* translation by facilitating duplex formation [24].

## 4. Regulation by RNA Binding Proteins

 The human genome is predicted to encode circa 1,000 RNA binding proteins (RBPs) with a large percentage of them implicated in translation. They could be categorized into two main groups: RBPs that are part of the basic translation machinery and required for the translation of all expressed mRNAs (examples: PABPI, elf4E) and RBPs that function in a more selective way by controlling either positively or negatively the levels of translation of specific target mRNAs (examples: HuR, Musashi1). Regarding this later group, it has been observed that RBPs can use distinct mechanisms to increase or inhibit translation. Although several exceptions are known, it can be said that RBPs often recognize specific motifs in UTRs and interact with the translation machinery to control expression. Interference with translation normally takes place during the initiation step (reviewed in [25]).

**Figure 2:**
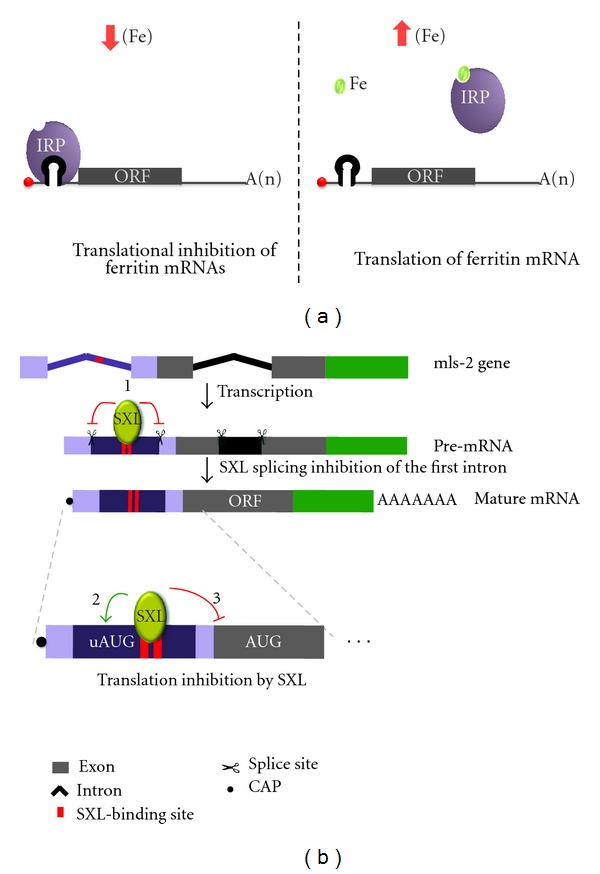
Translational regulation by RNA binding proteins. (a) In iron-deficient cells, IRPs bind to the IRE localized in the 5′ UTR of ferritin mRNA, blocking its translation. Once cellular iron levels increase, a complex containing Fe binds to IRPs. Thus, these proteins are allosterically modified, which reduces IRP-IRE binding and allows the translation of ferritin mRNAs. (b) *msl-2* gene regulation in females flies. After transcription in the nucleus, SXL specifically binds to intronic U-rich regions of msl-2 pre-mRNA and inhibits the intron removal (1). In the cytoplasm, SXL binds to the same elements localized now in the 5′ UTR of mature msl-2 mRNA, enhances the translation initiation of a upstream ORF (2), and prevents the main ORF translation (3). The regulatory elements in the 3′ UTR of msl-2 mRNA were not represented.

The best characterized example of RBP-mediated regulation involving 5′ UTRs is provided by the iron regulatory proteins (IRP 1 and 2). These proteins recognize a highly conserved stem loop structure with circa 30 nucleotides, known as the iron response element (IRE). The most important features include a hexanucleotide loop with the sequence CAGYCX (Y = U or A; X = U, C, or A) and a 5 bp upper stem that is separated from a lower stem of variable length by an unpaired cytosine. This regulation is crucial in maintaining cellular iron homeostasis as a large number of mRNAs connected to iron storage and metabolism including ferritin, mitochondrial aconitase, succinate dehydrogenase-iron protein, erythroid 5-aminolevulinate synthetase (eALAS), and an iron-exportin molecule named ferroportin (FPN1) have their expression modulated by this system. When cellular iron levels are low, IRP1 and IRP2 bind the IRE and block translation of the downstream ORF. When intracellular iron levels are high, the RNA binding activity of both IRPs is reduced (Figure 2(a)). IREs tend to be positioned close to the cap, which causes a steric inhibition of the binding of 40S ribosomal subunits to the transcript. When located distant to the cap, rather than affecting 40S recruitment, the IRE-IRP complex blocks ribosomal scanning (reviewed in [26]). An interesting bypass of the IRE/IRP mechanism can be observed in iron-starved duodenal and erythroid precursor cells. An upstream promoter is used to generate FPN1 pre-mRNAs containing one more exon that is connected by alternative splicing to a splice acceptor in the 3′ of the IRE. A mature FPN1 transcript containing the same open reading frame is generated; however, the 5′ UTR does not contain the IRE [27]. Therefore, these cells express the alternative FPN1 isoform in an iron-independent manner [27, 28]. Mutations affecting IREs can lead to diseases. This is the case of hereditary hyperferritinemia-cataract syndrome (HHCS), a genetic autosomal dominant disorder in which aggregation and crystallization of ferritin in the lens leads to bilateral cataracts [29].

 RBP-mediated regulation can be very elaborate and involve multiple steps. One good example showing the crosstalk between factors and distinct regulatory processes is the male-specific-lethal 2 (*msl-2*) gene in *Drosophila*, a main player in dosage compensation. The female-specific RNA binding protein sex lethal (SXL) participates in multiple aspects of *msl-2* regulation where *msl-2* expression must be prevented (Figure 2(b)). Regulation starts at the splicing level; SXL binds to two polyU stretches located in an intron that is part of the 5′ UTR. This process causes intron retention and preserves critical sequences that later will be used in translation regulation [30, 31]. In the cytoplasm, the same SXL protein will function as a translation repressor of *msl-2* in two distinct mechanisms taking place at the 3′ and 5′ UTR [32]. SXL binds U-rich sequences in the 3′ UTR and recruits the corepressor protein UNR (upstream of N-ras) and PABP blocking the recruitment of the pre-initiation complex to the 5′ end of the mRNA [33–35]. To assure that *msl-2* gets fully repressed, a second regulatory step also mediated by SXL takes place at the 5′ UTR. This repression involves a novel regulatory mechanism where crosstalk between SXL and a uORF takes place to efficiently repress translation [36]. The 5′ UTR of *msl-2* contains 3 uORFs but only the 3rd one is involved in the repression. Interestingly, this repression is very weak in the absence of SXL (~2-fold), but when present, SXL binds a poly U stretch a few nucleotides away from the uAUG and increases this repression to more than 14-fold. SXL acts by boosting translation initiation at the uAUG and not by acting as a simple steric arrest of scanning ribosomes. This effect may take place via an interaction between SXL and translation initiation factors; possibly members of elF3 component as indicated by a two-hybrid screening. This mechanism potentially affects a large number of mRNAs; 268 transcripts in *Drosophila* were determined to contain SXL binding motifs associated with uAUG spaced at an appropriate distance. For instance, a reporter construct containing the 5′UTR of the gene *Irr47 *was repressed ~4-fold by SXL protein [36].

 RBPs can have antagonistic functions when regulating translation. An interesting example is the regulation of p21 in the context of replicative senescence, a cellular state where cells enter an irreversible growth arrest. Induction of p21 is required to initiate the process, and to inhibit cdk2-cyclin E complexes. The 5′ UTR of p21 contains a GC-rich sequence that forms a stem loop. This element is recognized by two RBPs with distinct properties: CUGBP1 and calreticulin (CRT). Competition between the two proteins determines final levels of p21 expression and establishes if cells will proliferate or undergo growth arrest and senescence. Binding of CUGBP1 to p21 mRNA is dramatically increased in senescence compared to young fibroblast cells. Protein levels do not change during the process and this increase in activity is due to phosphorylation. On the other hand, CRT IPs showed a four-to-fivefold reduction of activity in senescence cells due to a decrease in expression. Both proteins were shown to affect p21 translation. However, while CUGBP1 functions as an activator, CRT acts as a repressor. Since the two proteins have opposing activity in senescent cells, they were examined to see if they compete for interaction with p21 mRNA and to control its translation. Increasing amounts of one protein were able to reverse the binding of the other protein to p21 mRNA and its effect on translation; affinity to the binding site is rather different as CUGBP1 had to be present in the binding reactions at a four-to-eightfold molar excess to CRT to antagonize its binding to p21 mRNA and impact its translation [37].

## 5. Regulation by uORFs and Upstream AUGs

 uORFs and uAUGs are major regulatory elements in 5′ UTRs. As their names suggest, uORFs are sequences defined by a start and stop codons upstream of the main coding region while uAUGs are start codons without an in-frame downstream stop codon located upstream of the main coding region. A large percentage of the human transcriptome contains uORF and/or uAUGs, with values ranging between 44 and 49% [38, 39]. Similar numbers are found in the mouse transcriptome. Although these numbers might sound high, both uORFs and uAUGs are less frequent than expected by chance, suggesting that they are under selective pressure. uORFs and uAUG are overrepresented in particular subgroups like transcription factors, growth factors, and their receptors and proto-oncogenes [6]. Both uORFs and uAUGs are extremely diverse varying in position in relation to the cap and main AUG, number per transcript and length (in the case of uORFs) [38]. Supplementary Table 1 (in Supplementary Material available online at doi:10.1155/2012/475731) provides a comprehensive list of uORFs and uAUGs present in the human transcriptome. uORFs and uAUGs have not been extensively analyzed in terms of conservation. A pilot study done with a subset of human, mouse, and rat transcripts indicated that both elements are moderately conserved as 38% of uORFs and 24% of uAUGs were determined to be conserved among three species [39]. The modest conservation of uORFs combined with the fact that their average length (20 nucleotides) is expected by chance and uAUGs provide a stronger suppression in comparison to uORFs suggests that many uAUGs have been neutralized in the process of evolution by the acquisition of a downstream stop codon. It has been proposed then that only a few uORFs, very likely the conserved ones, have been recruited for expression regulation [39]. In yeast, it has been shown that uORFs are statistically underrepresented in 5′ UTRs and were removed by selective pressure, indicating similarly that the remaining uORFs may be implicated in translation regulation [40].

**Figure 3:**
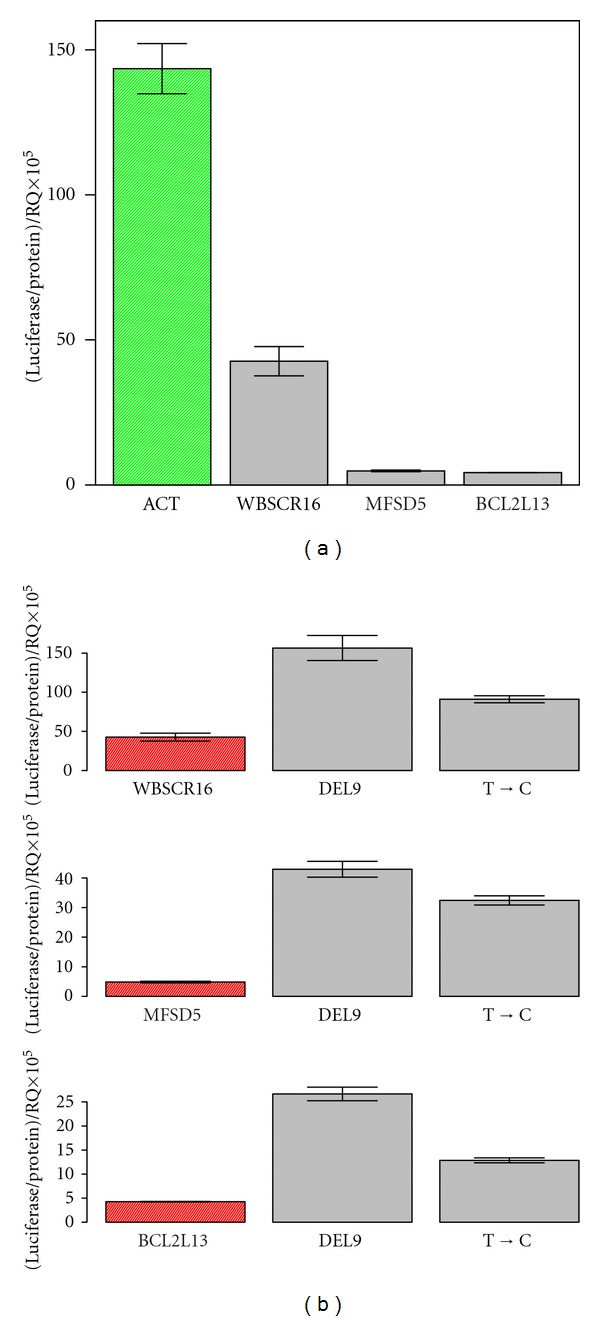
Impact of uAUG sequences on translation regulation. (a) Comparison of luciferase levels obtained for constructs having the 5′ UTR of the gene ACT (control) and genes containing uAUG: WBSCR16, MFSD5, and BCL2L13. (b) Deletion or mutation of uAUG sequence present in genes WBSCR16, MFSD5 and BCL2L13 reverts translation repression as seen as an increase in luciferase activity.

Although, overall it has been suggested that uORFs are negatively correlated with protein production [1, 38, 41] until now, functional activity has been demonstrated for only a limited number of uORFs and uAUGs. In Figure 3, we show examples of the impact uAUGs can have on translation efficiency. Among the most relevant features that can contribute to functionality are long 5′ cap-to-uORF distance, sequence conservation, context in which the AUG is located, strength of the initiation site for the ORF, length of the uORF, and number of AUGs in the 5′ UTR [38, 42]. Different outcomes have been observed when a ribosome encounters a uAUG or uORF [43]. Since the number of characterized events is still small, it is hard to define general mechanisms; we describe then a few well-characterized and relevant events. Leaky scanning is defined when a proportion of the scanning complexes bypass the uAUG or uORF and continue scanning for the next AUG. In this case, the upstream AUG acts as a “decoy” from the ORF AUG, functioning as a negative regulator of translation at least for some fraction of ribosomes. The production of cis-acting peptides by uORFs can reduce the initiation of translation of the downstream ORF by stalling the ribosome at the end of the uORF [44]. A classical example is provided by the evolutionarily conserved eukaryotic arginine attenuator peptide (AAP), that negatively controls the translation of proteins involved in the *de novo* fungal arginine biosynthesis in high arginine concentration [45]. In this scenario, arginine changes AAP conformation and/or P site environment causing ribosomal stalling at the termination codon of AAP uORF [46, 47]. AAP also reduces translation elongation by ribosome stalling when the uORF is inserted within an encoding sequence [48]. Another classical example of uORF-mediated regulation comes from yeast. Four uORFs are present in the 5′ UTR of the transcription factor GCN4. The first of the four uORFs is always efficiently translated regardless of the nutritional conditions. In unperturbed cells, rapid reloading of ribosomes and initiation cofactors allow translation of uORFs 2–4 while inhibiting the translation of the main ORF. In situations of amino acid starvation, initiation factors are scarce, resulting in a decelerated reloading of ribosomes and scanning across the sequences containing the uORFs. A functional initiation complex is reassembled only at the main coding sequence and GCN4 expressed. This mechanism allows a fast response to nutritional stress [49, 50]. Another similar example of regulated expression via uORF is the Carnitine Palmitoyltransferase 1C (CPT1C) gene. CPT1C regulates metabolism in the brain in situations of energy surplus. The presence of uORF in the 5′ UTR represses the expression of the ORF. However, this repression is relieved in response to specific stress stimuli like glucose depravation and palmitate-BSA treatment [51]. It has been suggested that uORFs can also induce mRNA degradation. A series of 5′ UTR constructs containing as a reporter the cat gene from the bacterial transposon Tn9 was tested in yeast. A single nucleotide substitution was used to create a 7-codon ORF upstream of the cat gene. The uORF was translated efficiently and caused translation inhibition of the cat ORF and destabilization of the cat mRNA [52]. A connection between uORFs and mRNA decay was also suggested based on a comparison between average levels of expression of uORF-containing and non-uORF-containing transcripts [41].

Several mutations that eliminate or create uORFs that end up altering protein levels have been connected to human diseases. Their relevance was discussed recently [53]. Predisposition to melanoma can be caused by a mutation that introduces a uORF into the 5′ UTR of the gene cyclin-dependent-kinase inhibitor protein (CDKN2A) [54]. Hereditary thrombocythemia is caused by a mutation that creates a splicing variant that eliminates a uORF, leading to an increase in protein production of the gene thrombopoietin [55]. Marie Unna hereditary hypotrichosis derives from a mutation that disrupts a uORF present in the 5′ UTR of the gene hairless homolog and consequently increasing its expression [56]. A transition from G to A in one of the uORFs present in the 5′ UTR of TGF-*β*3 transcript was determined to be associated with arrhythmogenic right ventricular cardiomyopathy/dysplasia (ARVC) [57]. Another group of five uORFs associated with diseases have been tested recently [58] using reporter assays; they include gonadal dysgenesis (*SRY*) [59], Van der Woude syndrome (*IRF6*) [60], Carney Complex Type 1 (*PRKAR1A*) [61], Hereditary pancreatitis (*SPINK1*) [62], and Thalassaemia-*β* (*HBB*) [63]. This list will certainly expand as more than 500 single-nucleotide polymorphisms (SNPs) creating or deleting uORFs have been reported.

## 6. Searching for Novel Regulatory Elements in the 5′ UTR

Only a small fraction of the posttranscriptional regulatory elements located in human 5′ UTRs have been characterized. Those identified UTR elements are catalogued in a web-resource maintained by Graziano Pesole's group called UTRdb (http://utrdb.ba.itb.cnr.it/) [49]. In vivo methods for the identification of posttranscriptional regulatory elements in UTRs, especially those associated with RBPs, have advanced dramatically in the last five years thanks to deep sequencing technology. CLIP and RIP-Seq are methods based on the isolation of RNA protein molecules (RNPs) via immunoprecipitation, followed by RNase digestion and precise identification of RBP binding sites with deep sequencing [64]. Although the number of RBPs analyzed so far by these methods is really small (reviewed in [65]), as deep sequencing technology becomes more accessible and the methods simplified, one could expect that very soon a large portion of the human RBP binding sites in UTRs will be mapped.

Another choice to map UTR elements regulating translation is to use purely computational methods based on analyzing the UTR sequences. These methods are based on identifying degenerate ribonucleotide patterns that have the expected properties of RBP binding sites. Similar methods have been applied for nearly 30 years to identify transcriptional regulatory in promoter sequences. These methods are reaching maturity, are very widely used, and have assisted greatly in compiling databases about transcriptional regulation (e.g., TRANSFAC) [66, 67]. Although much of the work directed towards designing and refining regulatory sequence analysis algorithms in context of transcriptional regulation can be adapted to corresponding analysis problems in the context of post-transcriptional regulatory elements, there are additional complications associated with RBP binding sites. The most obvious among these is that RBPs will have secondary structural preferences, and few existing analysis tools can incorporate information about RNA folding. Similarly, because of RNA folding regulatory elements can more easily function synergistically or display concerted binding to sequence elements that are distal in the primary sequence but very close in the folded molecule. Another difficulty is the lack of example translational regulatory elements for training the analysis. Based on a handful of well-studied examples, there is often a perception that RBP binding sites are on average shorter than transcription factors (TFs) binding sites, but this perception may be due to bias in the set of RBPs receiving the most research focus [65]. One of the most powerful methods for identifying regulatory elements is phylogenetic foot-printing, which takes advantage of locally elevated evolutionary conservation to reveal functional elements [5, 50, 51]. This logic works equally well for post-transcriptional regulatory elements. Unfortunately TF binding sites are also a major confound to direct application of computational sequence analysis for identifying 5′ UTR elements involved in translation. Elements involved in transcriptional regulation reside both up- and downstream of transcription start sites, and when 5′ UTRs are sufficiently short post-transcriptional regulatory elements are likely interleaved with TF binding sites.

Ultimately the best methods for identifying post-transcriptional regulatory elements will emerge from complementary application of experimental and computational techniques.

## Supplementary Material

We summarized the basic statistics for uORFs in human transcriptome (NCBI build37.3). Table 1.a shows the number of appearances of uORF-like sequences which contains AUG in 5′ UTRs in terms of mRNAs and genes. Also, we separated out two distinctive uORF-like sequences with matching termination codon in 5′ UTRs or without matching termination codon in 5′ UTRs. The detailed information for these two cases is listed in Table 1.b. Finally, Table 1.c shows how many uORF-like sequences are appeared on an individual 5′ UTR of each mRNA.
